# A method for determining local pulse wave velocity in human ascending aorta from sequential ultrasound measurements of diameter and velocity

**DOI:** 10.1088/1361-6579/aae8a0

**Published:** 2018-11-26

**Authors:** Madalina Negoita, Alun D Hughes, Kim H Parker, Ashraf W Khir

**Affiliations:** 1 Brunel Institute of Bioengineering, Brunel University London, London, United Kingdom; 2 Institute of Cardiovascular Science, University College London, London, United Kingdom; 3 MRC Unit for Lifelong Health and Ageing at UCL, London, United Kingdom; 4 Department of Bioengineering, Imperial College, London, United Kingdom; Ashraf.Khir@brunel.ac.uk

**Keywords:** ultrasound imaging, arterial stiffness, pulse wave velocity

## Abstract

*Background*: Pulse wave velocity (PWV) is an indicator of arterial stiffness, and predicts cardiovascular events independently of blood pressure. Currently, PWV is commonly measured by the foot-to-foot technique thus giving a global estimate of large arterial stiffness. However, and despite its importance, methods to measure the stiffness of the ascending aorta are limited. *Objective*: To introduce a method for calculating local PWV in the human ascending aorta using non-invasive ultrasound measurements of its diameter (*D*) and flow velocity (*U*). *Approach*: Ten participants (four females) were recruited from Brunel University students. Ascending aortic diameter and velocity were recorded with a GE Vivid E95 equipped with a 1.5–4.5 MHz phased array transducer using M-mode in the parasternal long axis view and pulse wave Doppler in the apical five chamber view respectively. Groups of six consecutive heartbeats were selected from each 20 s run based on the most similar cycle length resulting in three groups for *D* and three for *U* each with six waveforms. Each D waveform was paired with each U waveform to calculate PWV using ln(*D*)*U*-loop method. *Main results*: The diastolic portions of the diameters or velocities waveforms were truncated to allow the pairs to have equal length and were used to construct ln(*D*)*U*-loops. The trimmed average, excluding 10% of extreme values, resulting from the 324 loops was considered representative for each participant. Overall mean local PWV for all participants was 4.1(SD  =  0.9) m s^−1^. *Significance*: Local PWV can be measured non-invasively in the ascending aorta using ultrasound measurements of diameter and flow velocity This should facilitate more widespread assessment of ascending aortic stiffness in larger studies.

## Background

Cardiovascular diseases represent a major cause of death worldwide (Ezzati *et al* 2015). High blood pressure, hypertension, is a common risk factor for cardiovascular disease. Increased arterial stiffness especially the stiffening of the ascending aorta, contributes to hypertension, particularly in older people (Laurent *et al* 2001, Boutouyrie *et al* 2002). Furthermore, increased arterial stiffness predicts cardiovascular disease independently of blood pressure (Ben-Shlomo *et al* 2014). Currently, arterial stiffness is most commonly assessed by measuring pulse wave velocity (PWV), which, through Bramwell–Hill equation (Bramwell and Hill 1922), is inversely proportional to distensibility. Moens and Korteweg derived independently a related equation which expresses PWV in terms of vessel properties, i.e. proportional to the elastic modulus, and wall thickness, and inversely proportional to vessel radius and blood density (Tijsseling and Anderson 2012).

The most common technique for the non-invasive measurement of PWV employs the foot-to-foot technique to estimate PWV between the carotid and femoral arteries (Laurent *et al* 2001). Although the technique gives an estimate of aortic PWV, it cannot account for local variations in PWV due to the different dimensions and mechanical properties along the arterial tree.

More recently, techniques for assessing local arterial stiffness have been developed. A method using simultaneous measurements of pressure (*P*) and volumetric flow (*Q*) or velocity (*U*), or volumetric flow (*Q*), the PU-loop (Khir *et al* 2001, 2004) where the PWV is proportional to the ratio of the change in pressure to change in velocity across the wavefront, was introduced and tested in the canine aorta while occluding or not the aorta at different levels and observing the effect of distal occlusions at the level of the ascending aorta. For coronary arteries the sum of squares method was developed (Davies *et al* 2006, Aguado-Sierra *et al* 2006) to estimate PWV. Other methods have been introduced based on non-invasive measurements of area (*A*) and flow *Q* such as the *QA*-loop (Rabben *et al* 2004) which was tested, based on ultrasound measurements, both in human carotid and dogs; a method using diameter (*D*) and velocity (*U*) ln(*D*)*U*-loop (Feng and Khir 2010), derived from PU-loop and tested *in vitro*, in human carotid artery and canine aorta. Other methods based on the simultaneous measurement of pressure and area (the *D*^2^*P*-loop (Alastruey 2011)) have been suggested.

With the exception of the sum of the squares method (Davies *et al* 2006) all these techniques rely on the assumed absence of reflected waves in early systole. Techniques based on *D* and *U* have the advantage of collecting the measurements non-invasively using the same device. The ln(*D*)*U*-loop technique has been validated *in vitro* against invasive measurements (Li and Khir 2011) and has been shown to have good reproducibility (Pomella *et al* 2017). Although, the non-invasive ln(*D*)*U*-loop method was tested in carotid and femoral arteries of healthy adults (Borlotti *et al* 2012), its application in the ascending aorta of healthy humans using ultrasound measurements has not yet been widely used, and only recently, the feasibility of this technique was introduced and compared to carotid-femoral measurements (Negoita *et al* 2017).

The aim of this study was to describe a method of calculating local PWV in the adult ascending aorta non-invasively. The study describes the steps employed to make these calculations starting from acquisition of ultrasound images of *D* and *U*, as well as describing the optimization of offline analysis to extract the waveforms, align them based on the systolic upstroke for use in ln(*D*)*U*-loops to estimate PWV.

## Methods

### Study population

Ten healthy volunteers aged 22–32 years (four females) were recruited from students in Brunel University. The study was approved by the local ethics committee and written informed consent was obtained from each participant.

### Image acquisition

*D* and *U* were measured in the ascending aorta using a GE Vivid E95 ultrasound system with a 1.5–4.5 MHz phased array transducer. Participants were scanned in left-lateral decubitus position. *D* was recorded in the parasternal long axis view (PLAX) to ensure the measurements were perpendicular to the vessel center-line. M-mode was chosen in preference to B-mode to maximize the frame rate (Negoita *et al* 2016) and the M-mode cursor was placed immediately downstream of the sino-tubular junction. *U* was measured in the apical five chamber view (A5CH) using pulse wave (PW) Doppler with a 5 mm sample volume placed in the center of the aorta as close as possible to the location of the *D* measurement. A 3-lead ECG was recorded throughout all measurements and recordings lasting 20 s each were repeated three times with an interval of 1–2 min between measurements. Images were exported in DICOM format for offline analyses.

### Waveform extraction

**Figure 1.**
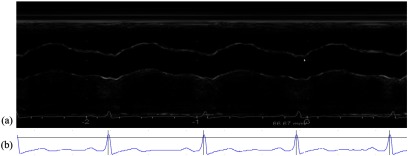
(a) Concatenated frames of a cine-loop of a M-mode recording used to determine *D* waveforms in a volunteer. (b) ECG signal as extracted from the concatenated image. The horizontal black line is the *R*-wave peak determined as the time of the maximum of the ECG following the crossing with the threshold of 0.8 * ECG height above which *R*-wave peaks are detected. The detection of the peaks is highlighted by the vertical lines.

As the images are renewed on the machine during acquisition, all the frames were concatenated (figure 1(a)) to ensure a continuous display of all the heart-beats. A semi-automatic Matlab code was developed in-house to extract the continuous waveforms of *U* and *D* of the ascending aorta. The code reads the DICOM images and the information about the acquisition was extracted from the DICOM header (image size, frame rate, etc). From each concatenated image, the ECG was also extracted (figure 1(b)). The *R*-wave of ECG was detected and the peaks of the QRS were used to separate the heartbeats. The frame rate was used to distinguish between the two acquisitions (*D* and *U*).

### Diameter waveform extraction

The scale is selected from the ultrasound image so that the final diameter waveform can be expressed in centimeters. The time axis, however is not rescaled into seconds (1 pixel is 3.5 ms).

**Figure 2.**
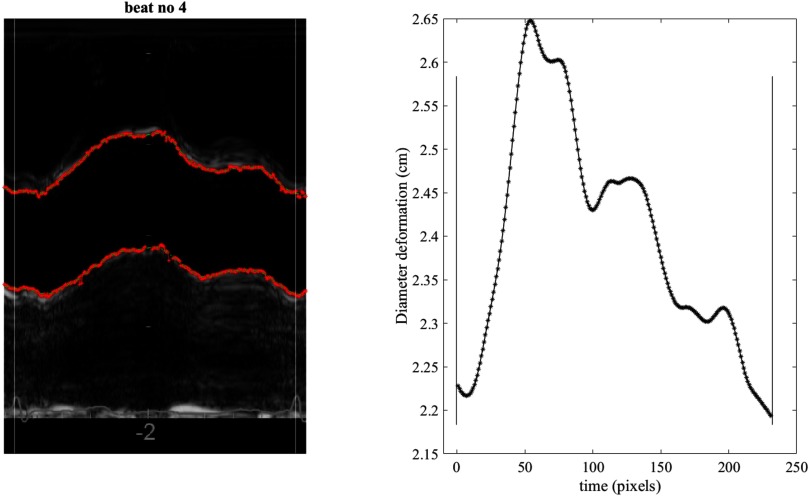
(a) A representative ultrasound image (M-mode) of the ascending aorta for one cardiac cycle. The tracing achieved by off-line analysis on the inner arterial walls is superimposed in red between two consecutive *R*-peaks as detected by the analysis and shown as vertical white lines. (b) The resulting diameter waveform calculated by subtraction of the two walls traced in (a). The waveform is shown between the same *R*-wave peaks (depicted by the black vertical lines). Time is shown in pixels where 1 pixel  =  3.5 ms.

To speed up the analysis and to optimize detection of the wall-lumen boundary during tracing, the user defined the areas of the posterior and anterior (or far and near) walls in which the thresholding was to take place, by clicking the image. To avoid including artefacts in the search for the wall edge, we used different starting positions for the top wall and bottom walls. Artefacts in the image were removed manually from the tracing and the threshold values for each wall were adjusted appropriately to ensure visually optimal tracing of the walls. A smoothing spline (smoothing parameter 0.001) was used to smooth the wall tracing as well as filling the gaps where no data points were found based on the threshold, or where artefacts were identified and removed. *D* was measured as the inner edge to inner edge of both walls (figure 2). Each cardiac cycle was analyzed separately. Extracted waveforms were saved as Matlab files to be used in the analysis and determination of PWV using the ln(*D*)*U*-loop method.

### Velocity waveform extraction

**Figure 3.**
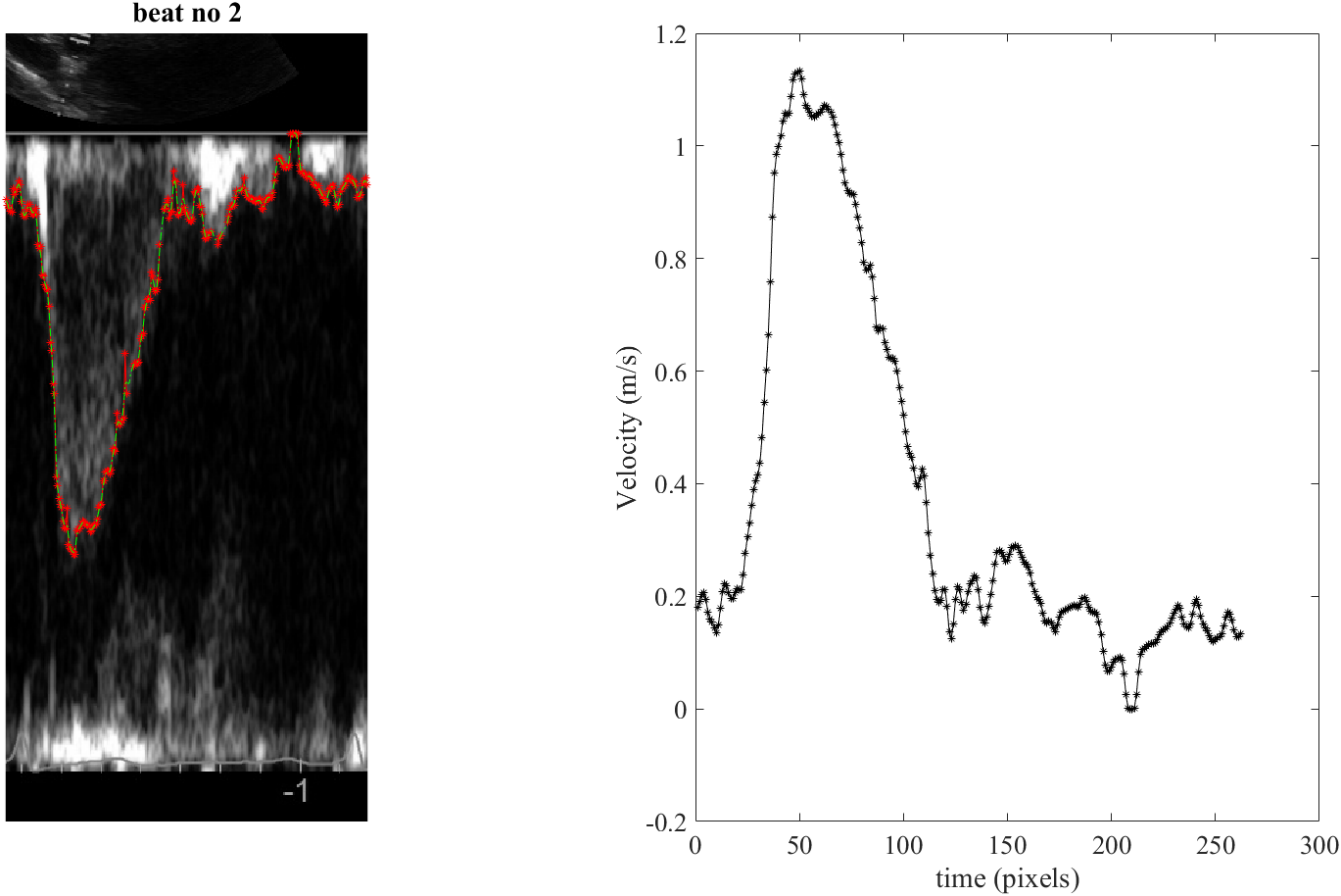
(a) A representative ultrasound image of the PW Doppler velocity for one cardiac cycle in the apical five chamber view (A5CH) view of the ascending aorta. The tracing achieved by off-line analysis (red points) is superimposed between two consecutive *R*-wave peaks. (b) The resulting velocity waveform extracted from the ultrasound image between the same two consecutive *R*-wave peaks.

For the extraction of the velocity waveform, a similar code based on grayscale image thresholding was used. The maximum Doppler envelope was traced to provide a measure of maximum velocity (Hoskins 2011). The scale was used for calibration and allows the conversion of *U* from pixels to m s^−1^. The start of the thresholding was manually selected. The threshold setting was selected by the user and, similar to the *D* waveform extraction, artefacts were excluded (figure 3). All waveforms were saved and flagged based on visual assessment of the quality.

### Determining the onset of upstroke

The onset of upstroke in both waveforms was defined as the data point after which the diameter and velocity waveforms begin to increase linearly in early systole due to ventricular ejection. To identify this point, first, the maximum and minimum of the waveform was determined. An automatic function starts in the middle of the systolic part and, going down point by point, calculated the goodness of fit of the linear regression of the segment, *r*^2^. When *r*^2^ became less than 0.985 the preceding point was taken as the onset of the upstroke. The onset of upstrokes in the D and U waveforms were used to time-align the ln(*D*)*U*-loops.

### Analyzed beats

M-mode (*D*) data were measured over three consecutive 20 s intervals; the transducer being removed from the chest wall and repositioned. The Doppler (*U*) velocity was then measured over three consecutive 20 s intervals following a similar protocol. For each run, all groups of six consecutive *D* and *U* waveforms were selected, with a moving window of one beat. The length (cycle duration) of each group is compared between all diameter and all velocity groups. The groups in each *D* and *U* that most closely corresponded in terms of heart rate (cycle duration) were selected as representative per run and used in further analysis.

Having selected a group of six diameter and six velocity waveforms per run, each *D* waveform in each group was combined with each *U* waveform in each group, to construct ln(*D*)*U*-loops. Selecting a number of waveforms as representative for the run allowed the user to visually inspect as well as correct the automatic determination of upstroke for the two waveforms and the linear fit of the ln(*D*)*U*-loop. The choice of six consecutive beats as the basis of our analysis is based on an informal comparison of the protocols used by a number of commercially available cardiovascular measurement devices (e.g. the SphygmoCor^5^5www.atcormedical.com.au/download/Active/Research_Manual_(CVMS).pdf.) which refreshes the acquisition every 5 s. For heart-rate of 60–80 bpm, 5 s will include 5–7 beats; hence our choice of using the average of six waveforms per run.

### Matching the *D* and *U* waveform cycle length

Due to the sequential acquisition of *D* and *U* and the variation in heart rate for the two acquisitions, the cycle length of the *D* and *U* waveforms could differ slightly. The results of ln(*D*)*U*-loop depend critically on the way the individual beats are paired. This could be done by pairing the beats with the most similar period or by pairing the beats with least noise. We, however, have taken a new approach of calculating the PWV for all possible pairings of the beats (6  ×  3*D*  ×  6  ×  3*U*  =  324 possible pairings) and estimating the PWV from the ensemble of the results for all possible pairings.

In calculating the ln(*D*)*U* loops it is essential that they are properly aligned in time. We do this by starting the ln(*D*)*U* loops at the onset of upstroke of both waveforms. Since the determination of PWV is based on the slope of the loop during early systole when it is assumed that only forward waves are present in the vessel, we ensure that the whole systolic portion of the loop is included by plotting the loop into the diastolic portion of the waveforms (in practice this is done by plotting the loop until the next peak of the *R*-wave in either waveform.).

The representative value per person is the mean of all the values obtained by matching individual beats. In order to exclude extreme values that might be due to a poorly extracted waveform, which in turn will lead to a poor ln(*D*)*U*-loop, a trimmed (truncated) mean was calculated by excluding the 10% of most extreme values.

### PWV determination

As previously reported (Feng and Khir 2010), in the absence of reflections in early systole, the relationship between *D* and *U* is expected to be linear and PWV is calculated as

1 \begin{align*} \newcommand{\e}{{\rm e}} \displaystyle {\rm PWV}=\pm\frac{1}{2}\frac{dU \pm}{d\ln (D) \pm}\nonumber \end{align*}

where *dU* is the change of diameter and *d*ln*D* is the change in the natural logarithm of diameter. The theoretical basis of equation (1) assumes a common onset of the upstroke of both diameter and velocity waveforms (as determined above). The fit of the ln(*D*)*U*-loop is automatic, commencing from the common point of onset of upstroke of both waves as determined above and including all adjacent points while *r*^2^  >  0.98. This threshold for *r*^2^ was chosen empirically to allow acceptable waveform analysis in the presence of the noise associated with the ultrasound images.

### Statistics

Statistical analysis was performed using Matlab. Data are presented as means and standard deviations (SD).

## Results

As explained earlier, ultrasound images of M-mode for *D* and A5CH for *U* are sufficient to give the parameters that construct ln(*D*)*U*-loops from which PWV can be estimated. The extraction of the waveforms is threshold-based, with an adaptive user-input threshold and visual assessment of the quality of waveforms.

Six consecutive heart beats are considered representative and sufficient to estimate PWV. From the repeated acquisition of three runs for diameters and three runs for velocities, each resulting in six waveforms, there are 324 possible pairings each giving a value of PWV.

**Figure 4.**
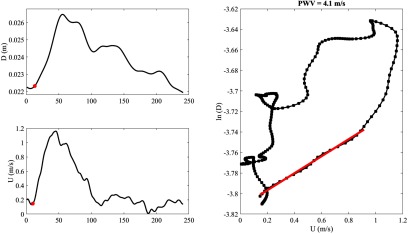
Representative examples of (a) a diameter waveform (b) a velocity waveform and (c) a ln(*D*)*U*-loop for the diameter and velocity shown in (a) and (b). The red line indicates the linear fit from which the pulse wave velocity (PWV) is calculated. In this example it is 4.1 m s^−1^. The red dots in (a) and (b) represent the onset of upstroke of the diameter and velocity waveforms used to align the start of the ln(*D*)*U*-loop in order to estimate PWV. Note that the ln(*D*)*U* loop is plotted for the entire cardiac cycle, although the determination of PWV depends only on the slope of the curve during early systole.

An example of a tracing of an M-mode ultrasound image and the extracted diameter waveform is shown in figure 2. Also, an example of a tracing and extraction of the velocity waveform from a PW Doppler ultrasound image is shown in figure 3. Figure 4 shows an example of the extracted *D* and *U* waveforms from the ultrasound images, and the ln(*D*)*U*-loop constructed using the two waveforms. The slope of the linear portion of the loop in early systole is determined and PWV is calculated using equation (1).

Across all participants analyzed, mean PWV was 4.1 (SD  =  0.9) m s^−1^. Individual values ranged from 3.2 (±0.5 within subject standard deviation (wsd)) m s^−1^ to 5.8 (±1.5 wsd) m s^−1^, with PWV of 4.0 (SD  =  0.7) m s^−1^ for females and 4.1 (SD  =  0.9) m s^−1^ for males. The individual values are presented in table 1.

**Table 1.** Individual pulse wave velocity (PWV) values obtained per volunteer as well as their age and gender. PWV data are means (±within subject standard deviations).

Volunteer	Gender	Age (years)	PWV (m s^−1^)
1	Male	22	3.8 ± 1.0
2	Male	23	3.4 ± 0.5
3	Male	24	3.7 ± 1.0
4	Female	24	5.1 ± 1.3
5	Female	25	3.2 ± 0.6
6	Male	28	3.1 ± 0.6
7	Male	28	5.6 ± 1.4
8	Male	29	4.9 ± 0.8
9	Female	30	4.0 ± 0.7
10	Female	32	3.7 ± 0.6

## Discussion

Local PWV in the aorta has been previously measured using MRI (Hickson *et al* 2010, Li *et al* 2010) or invasive techniques (Khir *et al* 2004) but this work presents a method that is ultrasound-based to determine PWV in the ascending aorta of human adults. We describe a technique for extracting the *D* and *U* waveforms from ultrasound images acquired with a commercial ultrasound system, which can be used with the ln(*D*)*U*-loop method to determine PWV non-invasively.

Ideally, ultrasound waves should be perpendicular to the vessel to measure diameter and in line with the flow to record flow velocity. Further, there are limited natural ultrasound windows to take the measurements in the ascending aorta. Together these make the simultaneous acquisition of velocity and diameter waveforms using ultrasound unfeasible.

Given that measurements were made consecutively, heart rate played a role into ‘size matching’ the *D* and *U* waveforms to construct the ln(*D*)*U*-loops where the waveforms need to have the same length. The truncating technique was chosen to estimate PWV since it removes a number of sampling points in late diastole to shorten one of the waveforms to size-match the other waveform. The method was preferred mainly to preserve the slope in systole of the waveforms, which is the period of interest for determining PWV. This might force the waveform to be different in late diastole from that of the original, but this is a tradeoff that does not affect our PWV determination. This approach was compared with other methods: interpolation and ensemble averaging. Although the average PWV was similar using different methods in our sample population (young healthy adults), this may not be the case when the method is used in older people or in patients with cardiovascular disease. The interpolation technique was dismissed due to a change in slope (i.e. PWV) when one waveform is extended to match the length of the other one. This would introduce major errors when the patient’s heart rate is not quasi-constant (e.g. atrial fibrillation patients) or even during exercise or recovery from exercise. Ensemble averaging the heart beats with different heart rates will lead to errors due to variability in isovolumic contraction time. Since the alignment is done with the use of *R*-wave peak, the onset of the waves will be different thus the averaging resulting in a slope slightly different than any individual beats. Thus, we suggest the use of the truncation method is most likely to provide a reliable estimation of PWV in the human ascending aorta.

## Limitations

Given the data acquisition, the analytical techniques and results presented in this work, it is feasible for this approach to be introduced into clinical practice. A limited training should allow any experienced sonographer to acquire the images; however some physical subject characteristics (e.g. obesity) may make the quality of images less than optimal. If the analytical technique can be further automated and integrated into commercial ultrasound scanner, the overall approach might be used as a screening tool, the subject of our future work.

The results of this study represent the average of three successive acquisitions, repeated on the same day with measurements taken approximately 1–2 min apart. In the view of the potential use of this technique as a clinical tool the reproducibility of the measurements has to be assessed, possibly over separate visits when the volunteer could be scanned anew.

The results presented in this study are based on a small number of young and healthy individuals. The narrow age range probably explains the lack of a clear correlation between local PWV and age. Future work is planned to include participants of all ages, genders and body shapes to establish reference values for local aortic PWV using this technique.

## Conclusions

PWV can be assessed in human ascending aorta using *D* and *U* waveforms to construct ln(*D*)*U*-loops. This approach shows promise as it is an easy, non-invasive technique, which is cheaper and more available than MRI. The technique can also assess the local material properties of the aorta using conventional clinical ultrasound machines.
